# Introduction to Advances in Sustainable Hydrogen Energy

**DOI:** 10.1039/d4ra90019g

**Published:** 2024-03-20

**Authors:** Nader Karimi, Larry K. B. Li, Manosh C. Paul, Mohammad Hossein Doranehgard, Freshteh Sotoudeh

**Affiliations:** a Queen Mary University of London UK n.karimi@qmul.ac.uk; b The Hong Kong University of Science and Technology Hong Kong larryli@ust.hk; c University of Glasgow UK Manosh.Paul@glasgow.ac.uk; d Texas A&M University USA doranehg@tamu.edu; e The Hong Kong University of Science and Technology Hong Kong mhd@connect.ust.hk; f University of Alberta Canada doranehg@ualberta.ca; g University of Houston USA fsotoudeh@uh.edu

## Abstract

Nader Karimi, Larry K. B. Li, Manosh C. Paul, Mohammad Hossein Doranehgard and Freshteh Sotoudeh introduce the *RSC Advances* themed issue on Advances in Sustainable Hydrogen Energy.
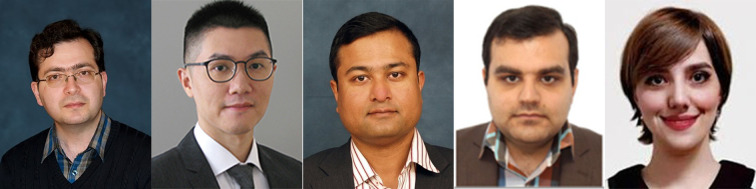

Developing a low-carbon economy is an essential prerequisite to sustainability. Despite an overwhelming desire for the replacement of fossil fuels, realisation of hydrogen economy continues to be a great challenge. Currently, there are a wide range of technologies for the production, storage, and utilisation of hydrogen. However, we are still yet to develop hydrogen based integrated energy systems that are economically viable and environmentally friendly.

Recently, there has been a new wave of research into system design and analysis which has greatly promoted the development of sustainable hydrogen energy. However, in order to complement elements of socioeconomics and policy making, we must also consider thermodynamic, technoeconomic, and environmental analyses in our research. Therefore, we were inspired to launch a themed collection dedicated to advancing sustainable hydrogen energy.

As the most environmentally friendly and cleanest fuel, hydrogen has the potential to replace the current fossil fuel-based energy infrastructure. In order to optimise hydrogen energy systems, it is important to understand the current status of achievable hydrogen energy. Le and Natsuki *et al.* establish an understanding of the current production, storage, and transportation methods, all within the context of the environment, economy, and society – this is imperative for developing a future outlook (https://doi.org/10.1039/D3RA05158G).

Hydrogen is produced from fossil-based or renewable sources, through various techniques such as thermochemical conversion, or biological conversion. Recently, there has also been progress in the use of metal organic frameworks (MOFs) for electrocatalytic water splitting. However, these methods are still not comparable to the commercial noble metal catalysts; therefore, further advancement is needed, such as the stabilisation of MOF materials during electrocatalysis, and the development of change-conducting MOFs (https://doi.org/10.1039/D3RA04110G).

There have been several applications of computational modelling techniques for the analysis of hydrogen energy systems. Simulation software allows for the creation of steady-state and dynamic models, so can be used to guide biomass gasification (a method of thermochemical conversion). Aspen Plus® enables accurate prediction of gasification behaviour, which in turn enables processing parameter optimisation (https://doi.org/10.1039/D3RA01219K). Mathematical modelling also allows for optimisation. Computational fluid dynamics (CFD) can be used to show the impact of throat sizing and gasification agents. Lower throat ratios result in higher yields and better gasifier performance, suggesting the potential for generation of CO_2_-free syngas (https://doi.org/10.1039/D3RA01408H).

Following on from modelling and optimisation, when assessing the feasibility of a large scale H_2_ gas production method, it is important to consider technoeconomic assessment. For instance, at optimal conditions, the eucalyptus wood sawdust (EWS) gasification process could be an appealing option for a hydrogen energy source in the market (https://doi.org/10.1039/D3RA00287J) – this ties in with the current focus on developing hydrogen generation technologies from renewable energy sources, particularly biomass.

Nevertheless, hydrogen storage is still a significant issue. Microwave-assisted hydrogen production could become an effective and cost-efficient method, especially given the potential for syngas transformation into high-hydrogen-content methanol, which would be easily storable and transportable (https://doi.org/10.1039/D3RA01898A). Though this method is still yet to face the challenges of large-scale production, such as parameter optimisation and economic assessment. Therefore, currently, pumped hydro storage (PHS), followed by compressed air technology (CAES) is the most common form of energy storage across the world. However, this method is heavily affected by geographical constraints so there are environmental concerns. Liquid air energy storage (LAES) could be an attractive alternative even though, commercially, this method is limited by its low efficiency and yields (https://doi.org/10.1039/D3RA04506D).

So, it is clear we must overcome a number of scientific, technological, and economic challenges in order to achieve the successful integration and hybridisation of hydrogen energy systems. This themed collection brings together the latest research findings in the rapidly growing field of hydrogen technology. We, the Guest Editors, would like to thank all the authors for the high quality of the contributions. We hope that these articles will be of interest to the multidisciplinary community of sustainable hydrogen energy and contribute to advancing the field of chemistry.

## Supplementary Material

